# Commensal nesting of White Storks (*Ciconia ciconia*), Spanish Sparrows (*Passer hispaniolensis*) and house sparrows (*Passer domesticus*)

**DOI:** 10.3897/BDJ.14.e184202

**Published:** 2026-04-16

**Authors:** Rusko Petrov, Tsvetina Dimitrova, Kristina Mironova

**Affiliations:** 1 Trakia University, Stara Zagora, Bulgaria Trakia University Stara Zagora Bulgaria; 2 Green Balkans - Stara Zagora NGO, Stara Zagora, Bulgaria Green Balkans - Stara Zagora NGO Stara Zagora Bulgaria

**Keywords:** *Ciconia ciconia*, *Passer hispaniolensis*, *Passer domesticus*, secondary nests, synanthropic species, breeding, biodiversity

## Abstract

This article presents the results of a study on a one-year-old nest of a pair of White Storks (*Ciconia ciconia*), emphasising its structure, biodiversity and the rescue operation following a traffic accident. The nest, located on an electric pole in the village of Bratya Kunchevi (Nova Zagora Municipality, Stara Zagora Region), developed into a complex synanthropic ecosystem including 151 secondary nests built by Spanish Sparrows (*Passer hispaniolensis*) and House Sparrows (*Passer domesticus*). The study revealed a significant number of eggs and nestlings of both sparrow species, alongside high losses due to mechanical damage and adverse environmental conditions. The results highlight the White Stork's role as an "ecosystem engineer" fostering urban biodiversity through synanthropic habitats, where sparrows commonly nest in stork structures. Analysis of the nest contents further quantified the high reproductive density within these structures, while also identifying significant mortality factors. Ultimately, the study concludes that stork nests serve as critical biodiversity hotspots in anthropogenic landscapes, providing essential nesting niches that support large, multi-species sparrow colonies. These findings underscore the importance of integrating conservation strategies for "engineer" species into rural infrastructure management to preserve these accidental, but vital ecosystems.

## Introduction

The White Stork (*Ciconia ciconia*) is a widespread species in Bulgaria, characterised by its high adaptability to anthropogenic environments. In recent decades, its nests have increasingly been found on electric poles, chimneys and other artificial structures, which often provide nesting sites for other bird species — most commonly sparrows. In Bulgaria and Europe, four sparrow species are found, three closely related: House Sparrow (*Passer domesticus*), Tree Sparrow (*Passer montanus*) and Spanish Sparrow (*Passer hispaniolensis*). The Spanish Sparrow has expanded its range over the last 40–50 years, while the Rock Sparrow (*Petronia petronia*) remains largely wild and avoids settlements. The transition of wild bird species into human settlements is a long historical process. Boev (1993) describes the archaeo-ornithological evidence for the synanthropisation of birds in the region, noting that sparrows were amongst the earliest species to adapt to human proximity. This deep historical association is supported by fossil records from the Chalcolithic and Medieval periods in Bulgaria, which show a consistent presence of passerines near human settlements (Boev 2025a, Boev 2025b).

**Figure 1.**
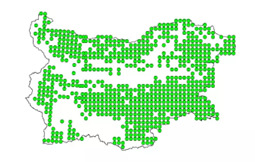
Distribution of the Spanish Sparrow in Bulgaria. (Bulgarian Society for the Protection of Birds – BSPB).

**Figure 2.**
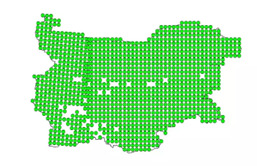
Distribution of the House Sparrow in Bulgaria (Bulgarian Society for the Protection of Birds – BSPB).

The Spanish Sparrow (*Passer hispaniolensis*) is a social, colonial species that prefers to build nests in sheltered spaces and frequently occupies the nests of larger birds. First recorded in Bulgaria in the early 20^th^ century, it is now widespread across the Thracian Plain and other parts of the country (Fig. 1). In some regions of Bulgaria, the Spanish Sparrow population is increasing at the expense of the House Sparrow, especially in rural and agricultural areas (Baumgart and Boev 2026). The House Sparrow most frequently nests under roof tiles, in building cavities, streetlight poles, tree hollows and nests of other birds, such as the House Martin (*Delichon urbicum*). Female Spanish Sparrows lay 4–5 eggs at 24–48-hour intervals, with incubation beginning after the penultimate or last egg is laid and lasting 11–14 days. Both parents participate in the care, with males often feeding incubating females (Bibby et al. 2000). Spanish Sparrow and House Sparrow are multi-brooded species that can breed repeatedly within a single breeding season when environmental conditions are favourable (BSPB 2025a, BSPB 2025b). The Spanish Sparrow's population has increased significantly in recent decades, often displacing the House Sparrow (Fig. 2). Environmental selection remains a main driver of divergence between these two species in the region (Geue et al. 2016). The Spanish Sparrow population in Bulgaria is estimated at 200,000 to 500,000 pairs, with the densest distribution in south-eastern Bulgaria. The largest colony in Europe, approximately 14,000 nests, is located near the village of Krushare, Sliven Region (Daskalova et al. 2007). Concerning habitats, the Spanish Sparrow is highly adapted to anthropogenic environments, frequently nesting in urban parks, gardens, villages, towns and industrial zones. A characteristic feature is its use of ready-made nests, predominantly those of the White Stork (Daskalova et al. 2007).

Sparrows are synanthropic species well adapted to human presence. They often nest in buildings, sheds, pole cavities and sometimes within the nests of larger birds, such as the White Stork, where they build secondary nests. The White Stork and sparrows typically co-exist peacefully, with the sparrows using the nests for protection and breeding (Kosicki et al. 2007, Tobolka 2011). Although cohabitation between storks and sparrows is well known, quantitative studies on these symbiotic nesting structures remain limited. This study aims to analyse the structure, biodiversity and breeding success of a secondary nesting colony developed within a White Stork nest that was partially destroyed by fire in August 2024 and later demolished in a traffic accident in 2025.

## Material and methods

The study was conducted in the village of Bratya Kunchevi, Nova Zagora Municipality, Stara Zagora Region, Bulgaria (42°24′N, 25°38′E). On 20 June 2025, a traffic accident occurred when a car struck an electric pole bearing an active nest of a White Stork pair. The nest had been built within a single breeding season (2025) and was part of an active colony comprising 151 nests documented in the area. In August 2024, the same nest had been partially destroyed by fire and, in 2025, it was demolished as a result of the traffic incident. The controlled dismantling of the nest structure and the rescue operation were carried out with the assistance of EVN Bulgaria – Electricity Supply, the Municipality of Stara Zagora and the Wildlife Rehabilitation and Breeding Centre of Green Balkans NGO, in accordance with methodological guidelines for monitoring stork nests in urbanised environments.

During the dismantling, all secondary nests of Spanish and House Sparrows were documented, including those containing eggs or newly-hatched chicks. Each nest was described in terms of height within the structure and degree of preservation. No adult individuals of any of the three species (storks, House Sparrows or Spanish Sparrows) were present. A total of four White Stork chicks were found and documented — two alive and two dead. The health status of the surviving individuals was assessed, following established protocols. A total of 132 sparrow nestlings were recorded — 96 alive and 36 dead. Morphological data were collected for all specimens following standard ornithological approaches (Cramp and Perrins 1994). A total of 28 eggs were placed in a specialised small-bird incubator at the Green Balkans Centre — 26 of these were fertilised and two were unfertilised. After incubation, 22 chicks successfully hatched; however, four died within the first seven days, consistent with typical early-period mortality rates.

Mortality was estimated as a percentage of the total number of sparrow nestlings observed in active nests. Causes of death were categorised into four groups: mechanical trauma, thermal stress, hypothermia and dehydration. The condition of the eggs was classified into four categories: healthy, fertilised, unfertilised and damaged. The developmental stage and condition of the nestlings were also analysed through direct in situ observations immediately after nest dismantling. All data were recorded photographically, by video and through measurements taken in accordance with standard ornithological protocols (Bibby et al. 2000).

## Results

The rescue operation for the young storks, as well as the chicks and eggs of the Spanish and House Sparrows, was conducted by the staff of the Green Balkans Wildlife Rehabilitation and Breeding Centre. The nest was dismantled using lifting equipment provided by EVN Bulgaria – Electricity Supply in Stara Zagora. A total of two live stork chicks were rescued, while 132 sparrow chicks were found — 96 alive and 36 dead. A total of 82 eggs were recorded, of which 54 were broken and too severely damaged to be classified and 28 were intact. The eggs and chicks originated from a mixed colony of House and Spanish Sparrows. Due to the nest falling during the traffic collision, the eggs and chicks were mixed. The chicks were naked and unfeathered, making it difficult to distinguish between the two species; thus, their numbers are presented collectively both for eggs and chicks.

### Condition of the Eggs and Hatching

**Figure 3.**
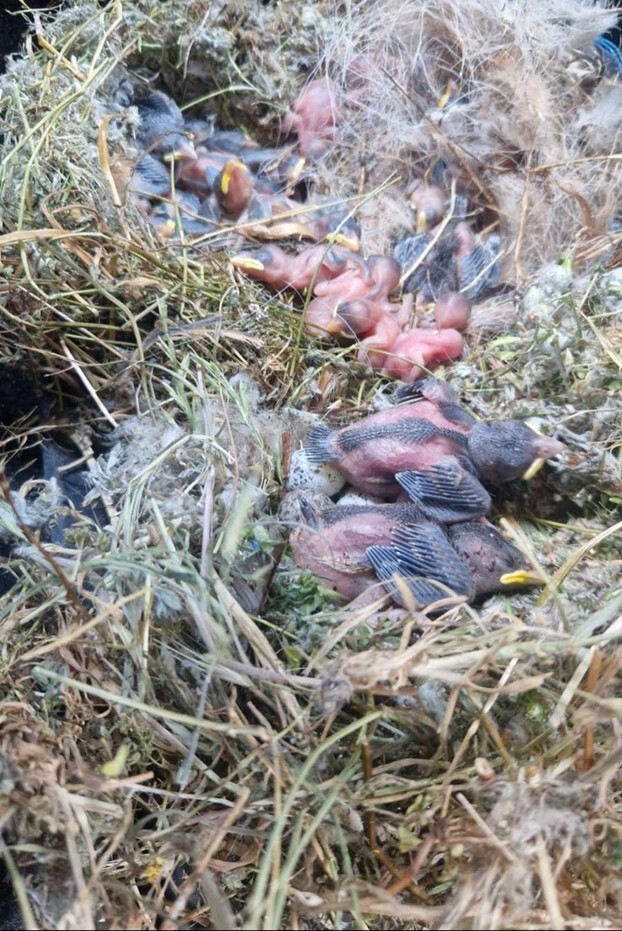
*In situ* cluster of sparrow nestlings and eggs within the nesting material immediately after the nest dismantling.

During the examination of the Spanish and House Sparrow nests, a total of 82 eggs were identified (Fig. 3). These were classified according to shell integrity into two primary categories: intact and damaged (Cramp and Perrins 1994). The morphology of the Spanish Sparrow’s eggs displayed distinctive characteristics separating the species from closely-related taxa. The eggs are relatively small, measuring 20–23 mm in length and 14–16 mm in width, making them smaller than those of the House Sparrow. Their shape is oval to slightly elliptical, with one end somewhat pointed. The colouration of the shell ranges from matte white to light beige, marked with spots in grey-brown, reddish or purplish hues, with denser pigmentation around the broader end (Cramp and Perrins 1994).

### Incubation and Survival

**Figure 4.**
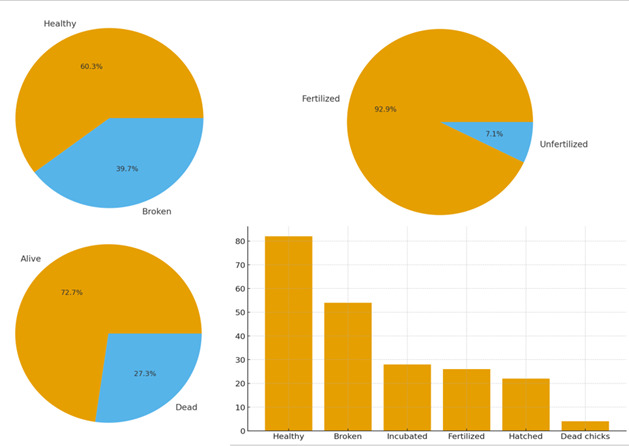
Percentage distribution of the main stages of incubation and survival. The diagram illustrates the proportion between different categories characterising reproductive success and survival. Of the total number of recorded categories, eggs constituted 22.0%, of which 4.5% were placed in an incubator and 4.2% were fertilised. The hatched chicks represented 3.6%, with 8.7% of all observed individuals being newly hatched. The proportion of healthy nestlings was 13.3%, while 15.5% of all recorded individuals were alive and 5.8% were dead. The total number of nestlings found amounted to 21.4%.

A total of 28 eggs were placed in a specialised small-bird incubator at the Green Balkans Centre — 26 of these were fertilised and two unfertilised. After incubation, 22 chicks successfully hatched; however, four died within the first seven days, consistent with typical early-period mortality rates (Table 1, Fig. 4).

### Assessment of Newly-hatched Nestlings of Passer hispaniolensis and Passer domesticus

**Figure 5.**
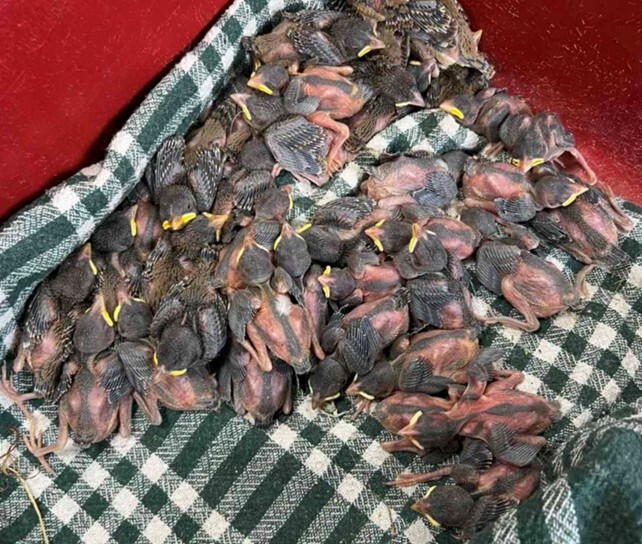
Aggregation of extracted nestlings (mixed species) placed on a heating cloth for triage and physiological assessment.

The nestlings of *Passer hispaniolensis* and *Passer domesticus* hatch in a completely altricial state, characterised by a high dependency on parental care and by poorly-developed integumentary structures (Starck and Ricklefs 1998). During the first 24 hours after hatching, the chicks are entirely naked, with thin, semi-transparent skin of pinkish to greyish hue through which subcutaneous capillaries are clearly visible. At hatching, the nestlings of Spanish Sparrow and House Sparrow are altricial, with closed eyes and poorly-developed limbs that are not capable of supporting the body. The bill appears relatively large and the gape margins are pale yellow to orange, which aids parental recognition during feeding (BSPB 2025a, BSPB 2025b) (Fig. 5).

During the first week after hatching, feather development in Spanish Sparrow and House Sparrow progresses from naked skin to the appearance of clearly visible feather follicles, observed as darkened points beneath the skin, particularly on the back and wings. This stage precedes the emergence of juvenile feathers enclosed in keratinous sheaths. At the same time, the skin gradually takes on a greyish colouration. Nestlings remain largely immobile with closed eyes, although the earliest coordinated reflex responses begin to develop (BirdLife International 2019,BSPB 2025a, BSPB 2025b).

Towards the end of the first week after hatching, some feather follicles elongate and the skin of Spanish Sparrow and House Sparrow nestlings begins to be partially covered by the developing juvenile plumage. During this phase of incomplete feathering, the chicks acquire a more advanced morphology, particularly in the brachial and lumbar regions, where the first contour feathers start to emerge from their keratinous sheaths (BirdLife International 2019,BSPB 2025a, BSPB 2025b). The eyes usually open around the 5^th^–6^th^ day and thermoregulation starts to develop, although still limited (Starck and Ricklefs 1998).

Between the second and third week after hatching, nestlings of Spanish Sparrow and House Sparrow become visibly feathered, although incomplete plumage coverage persists on the head and abdominal regions. Feathers remain relatively short, while the developing flight feathers on the wings and tail are clearly distinguishable. Exposed skin areas gradually darken to a greyish-brown tone, reflecting intensified growth processes during this phase. Behavioural changes are pronounced, with nestlings showing increasingly coordinated movements, attempts to stand and heightened responsiveness to external stimuli (BirdLife International 2019,BSPB 2025a, BSPB 2025b).

A total of 132 nestlings were recorded during the study period, of which 96 were alive and 36 were found dead. Observed mortality levels fall within the range typically reported for early developmental stages of altricial passerine species breeding in natural conditions in Bulgaria (WINGS Birding Tours Worldwide 2024,BSPB 2025a, BSPB 2025b).

**Figure 6.**
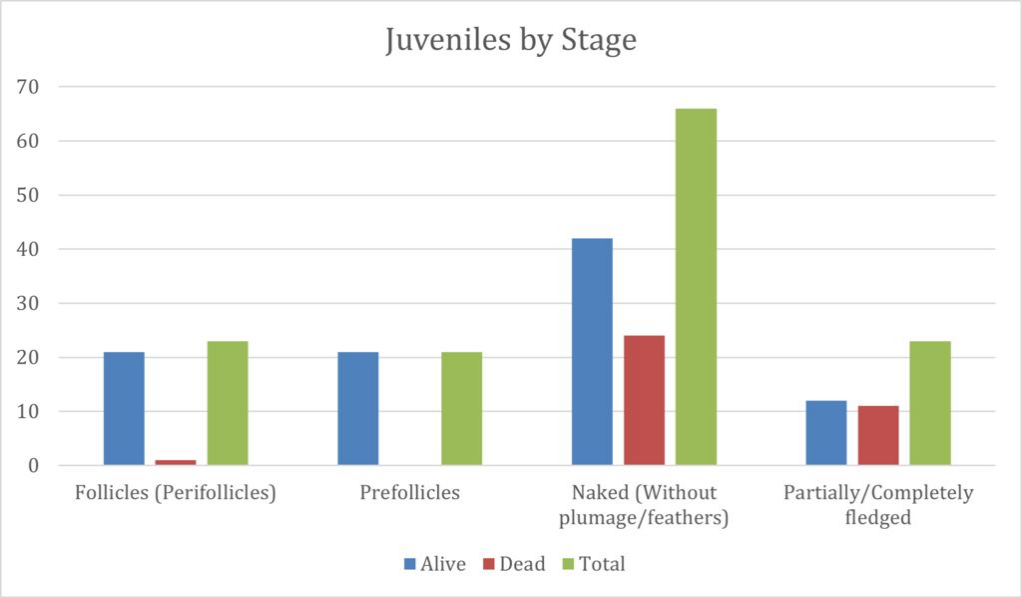
Distribution of the chicks by developmental stages. The graph presents the ratio between the total number of live, dead and total individuals at different stages of development: follicles (perfollicles), pre-follicles, naked (without feathers) and partially or fully feathered. The data are expressed as absolute numbers of individuals. A clear difference in abundance between the stages is observed.

Throughout the observations, the nestlings were classified according to the morphological development of the skin and plumage, with particular attention to the condition of the feather follicles (Grubb 1989). Nestlings classified in the early feather-development stages, characterised by the presence of emerging feather follicles, represent the initial and most active phases of plumage formation in Spanish Sparrow and House Sparrow, typically occurring within the first week after hatching. In contrast, completely naked nestlings constitute the most vulnerable developmental stage, exhibiting the highest mortality rates, which is consistent with patterns commonly observed in altricial passerine species under natural breeding conditions (BirdLife International 2019, WINGS Birding Tours Worldwide 2024, BSPB 2025a, BSPB 2025b). Partially or fully feathered individuals were more advanced, with closed or partially open eyes and improved thermoregulatory capacity, thereby increasing their chances of survival (Grubb 1989) (Table 2, Fig. 6).

## Discussion

Observed White Stork nests in Bulgaria function as secondary microhabitats, used not only by the storks themselves, but also by breeding colonies of Spanish Sparrow and House Sparrow. This highlights the role of certain species in creating or maintaining habitats that support other organisms. White Stork nests, therefore, provide important nesting and refuge sites for small synanthropic birds, particularly in rural and agro-urban landscapes (Boev 2024, WINGS Birding Tours Worldwide 2024,BSPB 2025a, BSPB 2025b).

The abundance of sparrows in such agricultural landscapes is well-documented. Dietary studies of the Barn Owl (*Tyto alba*) in southeast Bulgaria confirm that *Passer* species constitute a dominant prey item, reflecting their high population density and synanthropic success in the region (Milchev 2015). Furthermore, migration and wintering data indicate that, while some populations move, the availability of anthropogenic shelter (like stork nests) is crucial for resident survival (Nankinov 2014). The four species – *Falco tinnunculus* (Common Kestrel), *Asio otus* (Long-eared Owl), *Pica pica* (Eurasian Magpie) and *Passer hispaniolensis* (Spanish Sparrow) – were observed on a lone-standing Wild Pear tree, situated in the wild hacking area for Saker Falcons (*Falco cherrug*) in the Upper Thracian Lowland near Stara Zagora, Bulgaria. The availability of nests, combined with a rich food base, made this tree suitable for these species to utilise successfully in at least two breeding seasons without competition (Petrov and Pastir 2024).

The House Sparrow, in particular, shows a strong preference for microhabitats that mimic natural cavities, a trait well-documented in the breeding bird atlas of the region (BSPB 2025a). However, in this case, colonial nesting of *P. hispaniolensis* and *P. domesticus* was documented within a multi-layered structure, demonstrating a high degree of ecological plasticity and adaptation to anthropogenically altered environments. This phenomenon can be regarded as an example of facilitation — a positive interspecific interaction in which the structure built by one species supports the survival and reproduction of another (Bruno et al. 2003).

**Figure 7.**
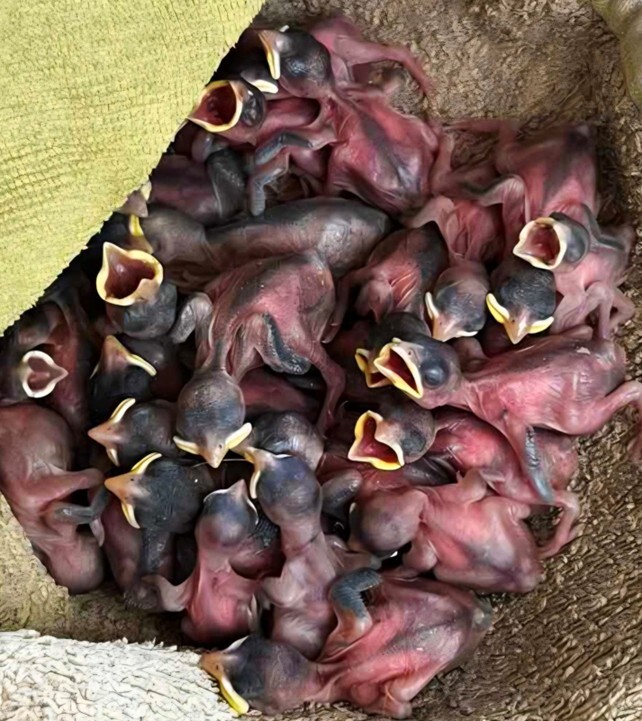
Close-up of nestlings showing varying stages of gaping behaviour (begging reflex) and skin vascularisation during the altricial phase.

The high proportion of broken eggs (65.9%) observed in Spanish Sparrow and House Sparrow nests was mainly caused by mechanical factors, such as the impact and collapse of the supporting electric pole. Physical disturbances of this type are known to result in significant egg loss in synanthropic bird populations. Additional contributing factors may include overcrowding within the nesting structure, leading to mechanical pressure and breakage from adult movements (BirdLife International 2019,BSPB 2025a, BSPB 2025b); vibrations and partial collapse of the nest, especially during rainfall and wind; predation or lack of parental supervision as a result of disturbance or partial destruction of the structure (Møller et al. 2015) (Fig. 7).

The highest mortality rates were recorded at the “naked” (36%) and “feathered” (47.8%) stages. Such values are typical for altricial species, whose early postnatal stages depend strongly on parental care and favourable microclimatic conditions (Starck and Ricklefs 1998). The primary causes of nestling mortality in Spanish Sparrow and House Sparrow include exposure to high temperatures and direct sunlight, resulting in thermal stress and dehydration; limited food availability, likely due to partial nest collapse or restricted access for parents; and occasional nestling abandonment, particularly following disturbances or behavioural changes in adult birds (WINGS Birding Tours Worldwide 2024,BSPB 2025a, BSPB 2025b). The combination of ecological and mechanical stress, together with the lack of parental care, likely resulted in the observed mortality levels.

These observations emphasise the need for more detailed research on the breeding success of small synanthropic species, such as Spanish Sparrow and House Sparrow, that utilise secondary habitats, as well as on the effects of human activities on their reproductive ecology (WINGS Birding Tours Worldwide 2024,BSPB 2025a, BSPB 2025b). This documented case confirms the importance of White Stork nests as secondary ecosystems with high biodiversity value. Observations in the village of Bratya Kunchevi demonstrate that a single stork nest can provide a viable microhabitat for over 150 individuals of Spanish Sparrow and House Sparrow, functioning as a multi-species habitat. The conducted rescue operation enabled not only the preservation of individuals, but also scientific study of the structure, materials and biology of the co-nesting species. The stork nest functions as a secondary habitat utilised by multiple small synanthropic species, confirming the role of the White Stork as an "ecosystem engineer" (Jones et al. 1994).

Spanish Sparrow and House Sparrow exhibit high ecological plasticity, adapting successfully to nesting conditions that differ from their typical natural habitats. The use of White Stork nests for colonial breeding reflects both behavioural and morphological flexibility (BirdLife International 2019,BSPB 2025a, BSPB 2025b). Effective institutional preparedness is essential for responding to incidents affecting urban or synanthropic bird nests. Coordination amongst municipal authorities, utility companies and conservation organisations is a crucial factor in minimising nest losses and enabling timely intervention (WINGS Birding Tours Worldwide 2024, BSPB 2025a, BSPB 2025b).

## Conclusions

This case emphasises the need for the development and implementation of emergency response protocols for incidents involving nests of synanthropic species and colonies of high conservation value. Such protocols should integrate systems for rapid localisation and assessment of threatened nests, standardised procedures for dismantling and relocating nests during emergency operations, training of power distribution and municipal teams in handling wild birds and cooperation with expert institutions, such as the Wildlife Rehabilitation and Breeding Centre of Green Balkans, which has extensive experience in artificial rearing, rehabilitation and release of birds. This integrated approach represents an effective model for sustainable co-existence between human infrastructure and wildlife conservation, especially in urbanised areas with a high concentration of synanthropic species.

For a deeper understanding of the ecological role of stork nests as secondary habitats, the following are recommended:

- Long-term monitoring of the breeding success of *Passer hispaniolensis* and *Passer domesticus*, as well as other secondary inhabitants of stork nests;
- Analysis of microclimatic parameters (temperature, humidity, ventilation) within the nesting structure and their influence on bird development;
- Investigation of the material composition and stability of nests to identify factors influencing the risk of collapse or fire;
- Behavioural observations of interactions between the host species and secondary inhabitants, particularly in the context of territoriality and interspecific tolerance;
- Development of management and prevention models for incidents involving stork nests, based on collaboration amongst energy companies, research institutions and conservation organisations.

## Figures and Tables

**Table 1.** Statistics of eggs and chicks.

Category	Number	Percentage of total eggs/chicks (%)
Total eggs	136	100%
— Healthy (intact)	82	60.3%
— Broken (damaged)	54	39.7%
Placed in incubator	28	—
— Fertilised	26	92.9% (of 28)
— Unfertilised	2	7.1% (of 28)
Hatched chicks	22	84.6% (of fertilised)
Dead chicks up to the 7^th^ day	4	18.2% (of hatched)
Total sparrows found	132	100%
— Alive	96	72.7%
— Dead	36	27.3%

**Table 2.** Classification of Chicks by Developmental Stage.

Stage	Alive (no.)	Dead (no.)	Total (no.)	Percentage of total (%)
Follicles (perfollicles)	21	1	22	16.7
Pre-follicles	21	0	21	15.9
Naked (without feathers)	42	24	66	50.0
Partially/fully feathered	12	11	23	17.4
Total	96	36	132	100
